# A scoping review protocol on food handlers’ knowledge, attitude, and practices towards food hygiene and safety in low and middle-income countries

**DOI:** 10.12688/f1000research.122822.1

**Published:** 2022-07-07

**Authors:** Paritosh Dabral, Senthil Kumaran Piramanayagam, Keith Nigli, Vijay Shree Dhyani

**Affiliations:** 1 Welcomgroup Graduate School of Hotel Administration (WGSHA), Manipal Academy of Higher Education, Manipal, Karnataka, India., India; 2 Public Health Evidence South Asia (PHESA), Department of Health Information, Prasanna School of Public Health (PSPH),, Manipal Academy of Higher Education, Manipal, Karnataka, India., India

**Keywords:** Food Hygiene, Food Safety, Food handlers, Knowledge, Attitude, Practices, KAP

## Abstract

Background: Food safety and hygiene has emerged as the foremost cause of concern in recent time, especially post-pandemic and has changed the eating out behaviour of the consumers. Consumers often consider food safety and hygiene as one of the most prominent factors and hence it is important for food handlers to have adequate knowledge and the right attitude towards food safety and food hygiene. The review will summarise the evidence on food handlers’ behaviours towards food safety and hygiene and associated factors that inhibit positive behaviour towards following food safety protocols and standards.

Methods: ****This scoping review protocol is guided by updated methodology from Joanna Briggs Institute (JBI). The search will be conducted on Medline (PubMed), Scopus and Web of Science. Google Scholar will be used to locate pertinent grey literature. A citation search will also be employed for identifying additional relevant studies. Quantitative and qualitative studies published from 2011- present will be included. Two reviewers will independently screen and extract the data. A third reviewer will be involved in resolving disagreements between reviewers. A two-stage screening including title/abstract and full-text will be conducted. Data extraction will be done using a pilot-tested data extraction form. The data extracted from included studies will be organised and presented using narrative synthesis. The review will also attempt to identify the unaddressed gaps in the literature with the available evidence.

Ethics and dissemination: An ethical clearance is not required for this scoping review as findings from existing published literature will be summarised. The review findings will be disseminated through conference presentations and journal publications.

## Introduction

Food is an integral and indispensable part of all cultures. However, illness—or even death—may result if contaminated food is consumed. Measures that prevent contamination of food during processing, preparation, and food handling thereby ensuring that the food is safe to consume are termed as food safety. “Any disease of an infectious or toxic nature caused by, or thought to be cause by, the consumption of food or water” is how the World Health Organization (WHO) defines foodborne diseases (FBDs). ^1^ FBDs continues to be the foremost public health challenge contributing to 420,000 mortalities and 600 million cases FBDs in 2010. ^2^ This makes the impact of FBD comparable to diseases such as malaria, tuberculosis, and HIV/AIDS. Although the complete economic and social impacts of FBD are not fully known, they are estimated to be enormous and considerable. ^3^ Instances of FBD have been reported from many countries around the world. LMICs have been hit particularly hard by instances of FBD ^4^^,^^5^ as access to clean water is scarce and the foodservice sector in these countries is mostly unorganised and informal. The Federation of Indian Chambers of Commerce and Industry (FICCI) in its report of 2017 ^6^ has indicated that about 66% of the foodservice establishments in India are in the unorganised sector. On the other hand, the possibility of wide-spread outbreak of FBD in high-income nations cannot be dismissed. In such countries, the highly consolidated structure of the foodservice industry with extensive and long supply chains has the potential to effect wide-spread occurrence of FBD as number of patrons frequenting such establishments is considerable.

The rise in eating out venues and accessibility to different cuisines are helping the food industry to post strong and sustained—except for a few periods of exception—growth for last two decades. ^7^ The dining-out behaviour has seen continuous increase due to factors such as convenience, higher income, working women, nuclear families, and globalisation. ^8^ The Indian domestic food service market is projected to be worth about INR 5 trillion with a CAGR of 10%. ^9^

Evidence from many research studies ^10^^–^^15^ suggest that food safety is an important consideration when selecting a dining-out destination. Czarniecka-Skubina, 2021 has reported an increased awareness amongst consumers about food safety and hygiene due to the ongoing COVID-19 pandemic. ^13^ Overall impression of the establishment, inspection ratings, grooming and attire of staff members, and observed cleanliness are the elements identified by Henson *et al.* (2006) that influence customers’ perception of an establishment’s food safety. ^16^ In their study, Sohn and Lee (2018) attribute the increase in number of restaurants and other foodservice operations that have an “open” kitchen design where patrons can observe the kitchen operations to patrons’ perception of offering better food quality and cleanliness. ^17^ Consequently such establishments are becoming popular amongst patrons who value food safety and cleanliness. ^17^^,^^18^

Many studies ^19^^–^^21^ identify insufficient among food handlers on Knowledge, Attitude and Practices (KAP) as the reason behind instances of FBD. A study by Knight *et al.* (2007) found that a majority of consumers believed that the food safety aspect at restaurants is significantly lower than—amongst others—grocery stores or supermarkets. ^10^ This perception of poor food safety at foodservice establishments in general and restaurants in particular has assumed significance as an instance of FBD at a foodservice business operation can have serious financial, societal, and economical implications. Grover and Dausch (2000) listed reduction in number of customers, adverse publicity, cost of litigation, and erosion of trust as the effects of a foodservice establishment being identified as the source of FB illness. ^22^

The instances of FBD are highest—3 out of every 5 cases—for food served in foodservice settings like restaurants, cafés, bistros etc. ^23^ Hedberg (2006) estimated that about 60% of these cases are due to unsafe food handling. ^24^ However, these figures are significantly lower than the actual number of cases as only about 7% of the customers who became ill due to FBD informed the health authorities. ^16^^,^^25^ The annual cost of illness in USA is about USD 6.9 billion for *top five* and between USD 51 to USD 77.7 billion for *all* foodborne pathogens. ^26^^,^^27^ This economic burden coupled with the top five factors recognised by FDA ^28^—poor personal hygiene, contaminated equipment, unsafe food sources, inadequate cooking, and incorrect holding temperature—for occurrence of FBD, indicate the significant contribution of foodservice establishments towards FBD and indicate the scope for limiting occurrences of FBD by engaging foodservice workers in the control of FBD by imparting relevant training.

Food hygiene practices such as keeping and cooking raw and cooked food separately at safe temperatures, using clean water for cooking are some of the ways to ensure food safety. ^29^ Thus it is important for food handlers to have adequate knowledge and right attitude towards food safety and they should also ensure practicing proper food hygiene and safety while handling the food. ^30^

Few systematic reviews of consumer’s Knowledge, Attitude and Practices (KAP) have been done previously. ^31^^–^^33^ However, there is scant and insufficient synthesis of various studies on KAP of food handlers. Thus, there is a necessity of synthesizing evidence on KAP of food handlers with regard to food safety and hygiene. To cater to this need, a scoping review of peer and non-peer-reviewed articles and grey literature is planned.

The objective of this review is to find out the KAP of food handlers in food facilities as well as potential factors affecting it. The review will also attempt to identify the unaddressed gaps in the literature.

## Methods

### Protocol design

Updated methodological guidance by JBI was followed for preparing the scoping review protocol. ^34^ Additionally, PRISMA Extension for Scoping Reviews ^35^ and scoping review methodology by Arksey and O’Malley’ ^36^ was also referred.

The scoping review will be conducted in five different stages.

### Identification of the research question

This review will address the following questions

(i) What is the level of food handlers’ KAP towards food hygiene and safety in LMICs?
(ii) What are the determinants that affects food handlers’ KAP in LMICs?
(iii) What are the gaps in the existing literature with respect to food handlers’ KAP?

### Study identification

**Inclusion criteria**

The English language articles from the year 2011 year onwards will be considered for this review. Population, concept, context (PCC) mnemonic will guide the inclusion/exclusion criteria for this review.

**Population**

The review will include food handlers above 18 years of age. Food handlers working in restaurants and in other food establishments such as street food handlers, food processing units, school canteens, food courts will be considered for inclusion. The review will also consider studies where consumer’s perception of food handler’s KAP is provided. Food handlers in hospital settings will be excluded.

**Concept**

Food hygiene and safety is increasingly assuming importance in today’s dining out environment. Customers are increasingly and critically examining the hygiene and safety scores—whether formal or informal—to determine their eating-out venue. Recognising this, many foodservice businesses are now getting their business audited and displaying scores prominently to attract customers. While examining factors that influence choice of eating-out venue; consumers often consider, food safety and hygiene as one of the most prominent factor. Thus it is very important for the food handler’s to have apt KAP and they should develop positive food handling behaviours. Some definitions related to the concept of this review are mentioned below.

“Food safety is about handling, storing and preparing food to prevent infection and help to make sure that our food keeps enough nutrients for us to have a healthy diet”. ^37^

“Food hygiene is defined as the measures and conditions necessary to control hazards and to ensure fitness for human consumption of a foodstuff taking into account its intended use”. ^38^

“A KAP survey is a quantitative method (predefined questions formatted in standardized questionnaires) that provides access to quantitative and qualitative information. KAP surveys reveal misconceptions or misunderstandings that may represent obstacles to the activities that we would like to implement and potential barriers to behaviour change”. ^39^

### Context

Studies from LMICs will be included in this review.

### Types of evidence sources

Studies involving different designs such as quantitative, qualitative and mixed method research designs will be considered for this review. We will also be including conference papers. However, letters to the editor, editorials, commentaries perspectives and reviews will be excluded.

### Search strategy

Studies published in English, conducted between 2011 and 2022 (both years included), and relevant to the review will be identified by conducting the search on Medline (PubMed), Global Index Medicus, Web of Science, Scopus, ProQuest and Google Scholar. The keywords guiding the search will include “Food handlers”, “Knowledge”, “Practices”, “Attitude”, “KAP”, “Food Hygiene, “Food Safety”, “Restaurant”, “Food safety training”, “Food Hygiene training”, “Food service establishment”, “Behavior”, “Facilitators, “factors” “and “Barriers”. PCC mnemonic described in JBI methodology will guide the search. Also, the references of included articles included in the review will be hand searched to locate and include any relevant study that meet the inclusion criteria. The preliminary search conducted on PubMed ( Table 1) combining keywords with Boolean operators will be refined and customized for other databases in consultation with the librarian.

**Table 1.** Preliminary Search Strategy for PubMed.

Concept	Key words and queries
Knowledge, Attitude and Practices (KAP)	((((((((knowledge)) OR (attitude)) OR (practices)) OR (KAP)) OR (“Knowledge, Attitude and Practices ”)) OR (“food safety behavior”)) AND ((((((“Food safety”) OR (“Food hygiene”)) OR (“food safety and hygiene”))) OR (“service hygiene”)) OR (“safety procedures”)))
AND	
Food Service establishments	((((((restaurants) OR (“food service establishment”)) OR (“food processing units”)) OR (canteens)) OR (“food courts”)) OR (“food establishments ”))
Filters:	from 2011-2022

### Selection of eligible studies

All the citations from the databases search will be imported to Rayyan software. Records after removing duplicates will undergo title, abstract and full-text screening. A two stage identical screening process will be followed, where every article will be independently reviewed by two reviewers following pre-defined inclusion and exclusion criteria. A consensus approach will be used to resolve discrepancies among the reviewers. If the disagreement cannot be resolved this way, a third reviewer will be asked to adjudicate. Conclusively relevant sources will be retrieved in full. The selected full text will be reviewed in minute detail against the eligibility criteria independently. In case of further ambiguities about the eligibility of an article, it will be labelled and discussed with the third reviewer. Search results indicating the number of articles included and excluded at different screening stage and exclusion reasons at full text screening stage will be depicted in PRISMA flow chart. ^40^

### Data extraction and charting

A piloted data extraction form will be used to collect the data relevant to research questions. Specific information on citation details, methodological characteristics and associated outcome measures of significance will be included in data extraction form. To ensure that the data is adequately extracted, the form will be pilot tested before final data extraction starts. Modifications will be incorporated as a result of piloting the extraction and will be documented in the scoping review. Two reviewer will independently extract the data from included studies. The discrepancies between reviewers will be discussed with a third reviewer. A request will be made to the authors of studies included in the review to obtain the required missing information. Preliminary data extraction/charting sheet is presented in Table 2.

**Table 2.** Data extraction/charting sheet.

Item	Description (including examples of categories, which will be extended based on included studies)
**Name of reviewer**	
**Citation information**	
Study title	
Study ID (Authors, Publication year)	
Journal name	
Author affiliation	
Contact information	
Inclusion criteria (all must be present)	
**Study characteristics**	
Aim/Objectives of the study	
Study period	
State, Country	
Study design	
Target Population	
Years of data collection	
Sample size	
Sampling method	
Study Perspective	
Setting	
Data Sources	
KAP Scores	
Correlation between knowledge, attitude and practices	
Factors associated with food handling knowledge, attitude and practices	
Key findings	
Conclusion	

### Stage 5: Data analysis and reporting

Data extraction will be followed by a narrative summary supported by descriptive statistics. Synthesising data will involve study characteristics such as study type, participant characteristics, setting, results, outcome variables etc. Quantitative studies will be analysed using descriptive statistics, such as frequencies, percentage, measures of central tendency and standard deviation. Analysis will be conducted based on population (food handlers), study designs, theoretical approaches and methods, geographical location, year wise distribution of the studies, association measured with respect to KAP, factors associated with KAP and food service setting. Moreover, for qualitative studies, thematic analysis will be assimilated for the analysis.

## Discussion

This will be the first scoping review to provide aggregate summary of evidence on food handlers’ knowledge, attitude and practices in LMICs. It will map the coverage of the evidence available and existing gaps on the topic. The scoping study will employ a comprehensive search strategy which will be conducted on five electronic databases. Grey literature search on Google Scholar and citation search will also conducted to include all possible relevant articles. However, there are limitations to the search with respect to the language and number of databases covered. Non-English articles will be excluded due to lack of resources (human and monetary) to translate in English. Quality assessment of the included in scoping review will not be undertaken as it is not the mandatory requirement of the scoping review.

### Study status

The review is in its initial phase of conceptualisation wherein the research question and eligibility criteria have been finalised. Currently team is working on finalising the search terms and search strategy.

## Data availability

No underlying or extended data are associated with this article.

## Reporting guidelines

Figshare. PRISMA-P checklist. DOI: https://doi.org/10.6084/m9.figshare.20055176.v1

## Author contributions

PD is the guarantor of this review protocol. PD, SK, KSN and VSD contributed to the title and concept genesis. PD drafted the protocol manuscript. All the authors have critically reviewed, proofread and given the final approval of the protocol version to be published.

## References

[1] Schmidt K : WHO surveillance programme for control of foodborne infections and intoxications in Europe. Sixth Report 1990-1992. 1995.

[2] WHO: *WHO estimates of the global burden of foodborne diseases: foodborne disease burden epidemiology reference group 2007-2015.* Geneva PP - Geneva: World Health Organization;Accessed 22 Dec 2021. Reference Source

[3] Devleesschauwer B Haagsma JA Mangen M-JJ : *The Global Burden of Foodborne Disease BT - Food Safety Economics: Incentives for a Safer Food Supply.* Roberts T , editor. Cham: Springer International Publishing;2018; p.107–122. 10.1007/978-3-319-92138-9_7

[4] UNICEF, WHO: Health in the post-2015 development agenda: need for a social determinants of health approach: joint statement of the UN Platform on Social Determinants of Health. In: Health in the post-2015 development agenda: need for a social determinants of health approach: joint statement of the UN Platform on Social Determinants of Health. 2013; p.18.

[5] Käferstein F : Foodborne diseases in developing countries: aetiology, epidemiology and strategies for prevention. *Int. J. Environ. Health Res.* 2003 Jun;13 Suppl 1:S161–S168. 12775392 10.1080/0960312031000102949

[6] Jaisani L Shukla A Malik R : Indian food services industry: engine for economic growth & employment. A Rep Unlocking Growth Oppor New Delhi FICCI. 2017.

[7] Naska A Katsoulis M Orfanos P : Eating out is different from eating at home among individuals who occasionally eat out. A cross-sectional study among middle-aged adults from eleven European countries. *Br. J. Nutr.* 2015 Jun;113(12):1951–1964. 10.1017/S0007114515000963 25907775

[8] Rezende DC de Avelar AES de : Factors that influence the consumption of food outside the home in Brazil. *Int. J. Consum. Stud.* 2012 May 1;36(3):300–306. 10.1111/j.1470-6431.2011.01032.x

[9] KPMG: *India’s Food Service Industry: Growth Recipe.* KPMG and FICCI;2016.

[10] Knight AJ Worosz MR Todd ECD : Serving food safety: Consumer perceptions of food safety at restaurants. *Int. J. Contemp. Hosp. Manag.* 2007;19:476–484. 10.1108/09596110710775138

[11] Liu P Lee YM : An investigation of consumers’ perception of food safety in the restaurants. *Int. J. Hosp. Manag.* 2018;73:29–35. 10.1016/j.ijhm.2018.01.018 Reference Source

[12] Jones TF Angulo FJ : Eating in restaurants: a risk factor for foodborne disease? *Clin. Infect. Dis. Off. Publ. Infect. Dis. Soc. Am.* 2006 Nov;43(10):1324–1328. 10.1086/508540 17051501

[13] Kim HJ Park J Kim M-J : Does perceived restaurant food healthiness matter? Its influence on value, satisfaction and revisit intentions in restaurant operations in South Korea. *Int. J. Hosp. Manag.* 2013;33:397–405. 10.1016/j.ijhm.2012.10.010 Reference Source

[14] Wei Y-P : The Effect of Food Safety-Related Attributes on Customer Satisfaction of Ready-to-Eat Foods at Hypermarkets. *Sustainability.* 2021;13. 10.3390/su131910554

[15] Ungku Fatimah UZA Boo HC Sambasivan M : Foodservice hygiene factors—The consumer perspective. *Int. J. Hosp. Manag.* 2011;30(1):38–45. 10.1016/j.ijhm.2010.04.001 Reference Source

[16] Henson S Majowicz S Masakure O : Consumer assessment of the safety of restaurants: The role of inspection notices and other information cues. *J. Food Saf.* 2006;26(4):275–301. 10.1111/j.1745-4565.2006.00049.x

[17] Sohn E-M Lee K-W : The effect of chefs’ nonverbal communication in open kitchens on service quality. *J. Foodserv. Bus. Res.* 2018;21(5):483–492. 10.1080/15378020.2018.1459125

[18] Chow AJ Alonso AD Douglas AC : Exploring open kitchens’ impact on restaurateurs’ cleanliness perceptions. *J. Retail. Leis. Prop.* 2010;9(2):93–104. 10.1057/rlp.2009.26

[19] Baş M Şafak Ersun A Kıvanç G : The evaluation of food hygiene knowledge, attitudes, and practices of food handlers’ in food businesses in Turkey. *Food Control.* 2006;17(4):317–322. 10.1016/j.foodcont.2004.11.006 Reference Source

[20] Vitória AG da Souza Couto Oliveira J de Almeida Pereira LC de : Food safety knowledge, attitudes and practices of food handlers: A cross-sectional study in school kitchens in Espírito Santo, Brazil. *BMC Public Health.* 2021;21(1):349. 10.1186/s12889-021-10282-1 33579231 PMC7881630

[21] Al Banna MH Disu TR Kundu S : Factors associated with food safety knowledge and practices among meat handlers in Bangladesh: a cross-sectional study. *Environ. Health Prev. Med.* 2021;26(1):84. 10.1186/s12199-021-01004-5 34454422 PMC8403395

[22] Grover SF Dausch JG : Hepatitis A in the foodservice industry. *Food Manage.* 2000;81:80–86.

[23] Lynch M Painter J Woodruff R : Surveillance for foodborne-disease outbreaks–United States, 1998-2002. *Morb Mortal Wkly report Surveill Summ (Washington, DC 2002).* 2006 Nov;55(10):1–42. 17093388

[24] Hedberg CW Smith SJ Kirkland E : Systematic environmental evaluations to identify food safety differences between outbreak and nonoutbreak restaurants. *J. Food Prot.* 2006 Nov;69(11):2697–2702. 10.4315/0362-028X-69.11.2697 17133814

[25] Green LR Selman C Scallan E : Beliefs about meals eaten outside the home as sources of gastrointestinal illness. *J. Food Prot.* 2005 Oct;68(10):2184–2189. 10.4315/0362-028X-68.10.2184 16245727

[26] Crutchfield SR Roberts T : Food safety efforts accelerate in the 1990’s. *FoodReview.* 2000 Jan 1;23(3):44–49. Reference Source

[27] Scharff RL : Economic burden from health losses due to foodborne illness in the United States. *J. Food Prot.* 2012 Jan;75(1):123–131. 10.4315/0362-028X.JFP-11-058 22221364

[28] Administration USF D. : FDA report on the occurrence of foodborne illness risk factors in selected institutional foodservice, restaurant, and retail food store facility types. *Public Heal. Serv.* 2009.

[29] Prevention C for DC and. Four steps to food safety: clean, separate, cook, chill. 2020.

[30] Akabanda F Hlortsi EH Owusu-Kwarteng J : Food safety knowledge, attitudes and practices of institutional food-handlers in Ghana. *BMC Public Health.* 2017;17(1):40. 10.1186/s12889-016-3986-9 28061850 PMC5219779

[31] Young I Thaivalappil A : A systematic review and meta-regression of the knowledge, practices, and training of restaurant and food service personnel toward food allergies and Celiac disease. *PLoS One.* 2018 Sep 4;13(9):e0203496. 10.1371/journal.pone.0203496 30180206 PMC6122805

[32] Young I Waddell L : Barriers and Facilitators to Safe Food Handling among Consumers: A Systematic Review and Thematic Synthesis of Qualitative Research Studies. *PLoS One.* 2016 Dec 1;11(12):e0167695. 10.1371/journal.pone.0167695 27907161 PMC5132243

[33] Zanin LM Cunha DT da Rosso VV de : Knowledge, attitudes and practices of food handlers in food safety: An integrative review. *Food Res. Int.* 2017 Oct;100(Pt 1):53–62. 10.1016/j.foodres.2017.07.042 28873718

[34] Peters MDJ Marnie C Tricco AC : Updated methodological guidance for the conduct of scoping reviews. *JBI Evid. Synth.* 2020 Oct;18(10):2119–2126. 10.11124/JBIES-20-00167 33038124

[35] Tricco AC Lillie E Zarin W : PRISMA Extension for Scoping Reviews (PRISMA-ScR): Checklist and Explanation. *Ann. Intern. Med.* 2018 Oct;169(7):467–473. 10.7326/M18-0850 30178033

[36] Arksey H O’Malley L : Scoping studies: towards a methodological framework. *Int. J. Soc. Res. Methodol.* 2005 Feb 1;8(1):19–32. 10.1080/1364557032000119616

[37] FAO: Food safety.Accessed 22 Dec 2021. Reference Source

[38] Holah J Lelieveld H : *Hygienic design of food factories.* Elsevier;2011.

[39] Gumucio S Luhmann N Fauvel G : The KAP survey model: knowledge, attitude, and practices. *Saint-Etienne Fr.* 2011;4–5.

[40] Page MJ McKenzie JE Bossuyt PM : The PRISMA 2020 statement: an updated guideline for reporting systematic reviews. *BMJ.* 2021;372:n71. 10.1136/bmj.n71 33782057 PMC8005924

