# Supplementary material for: A scoping review protocol on food handlers’ knowledge, attitude, and practices towards food hygiene and safety in low and middle-income countries
Source: F1000Res. 2022 Jul 7;11:757. [Version 1] doi: 10.12688/f1000research.122822.1 (PMC9493400; doi:10.12688/f1000research.122822.1)
Supplement: Peer review history

# Peer review: Reviewer response for version 1

**DOI:** 10.5256/f1000research.134861.r147956

**Published:** 2022-09-21

**Authors:** Hibbah Osei-Kwasi

**Affiliations:** 1 Loughborough University, Loughborough, UK

Thank you for the opportunity to review this paper focusing on a very important topic. As a scoping review, the methodology is transparent and robust and makes reference to existing guidelines which is always good practice. 

 I have a few minor suggestions:

- Can the authors provide a justification for the time frame for the searches? Why focus on that range?
- I also recommend including the LMIC countries in the search strategy.
- Also, the paper will benefit from a final proofread. A few points include consistency in some words: e.g. amongst/among... Authors should not mix British with American English.
- Finally, can authors check the in-text citation for consistency as well.

Is the study design appropriate for the research question?

Yes

Is the rationale for, and objectives of, the study clearly described?

Yes

Are sufficient details of the methods provided to allow replication by others?

Yes

Are the datasets clearly presented in a useable and accessible format?

Not applicable

Reviewer Expertise:

Nutrition, Public health, systematic reviews.

I confirm that I have read this submission and believe that I have an appropriate level of expertise to confirm that it is of an acceptable scientific standard.

# Peer review: Reviewer response for version 1

**DOI:** 10.5256/f1000research.134861.r147952

**Published:** 2022-09-01

**Authors:** Christopher J Bailey

**Affiliations:** 1 College of Education, Psychology and Social Work, Flinders University, Adelaide, Australia

The authors provide a scoping review protocol on food handlers' knowledge, attitude, and practices towards food hygiene and safety in low and middle-income countries.

 The paper is well-written and methodologically sound. The authors have identified an important research gap in the literature. The reviewer has only two questions for the authors:

1. On page 5 of the Data extraction and charting section, the authors state: “to ensure that the data is adequately extracted, the form will be pilot tested before final data extraction starts." It could be possible that the authors might identify significant large numbers of papers as they have included qualitative, quantitative, mixed methods, and grey literature.
2. The following question relates to if a large number of papers are identified - how many abstracts will the authors be extracting before data extraction? Will there be an agreed agreement or percentage for agreement, e.g., Kappa agreement >0.80 for relevant screening? Will there be a search string used for Google Scholar? (e.g., Food handlers’ knowledge, attitudes, and practices)

The reviewer has provided a reference regarding best practices for using Google Scholar and grey literature for grey literature. Although the author Haddaway *et al.* (2015) ^1^ discusses how the role of Google Scholar is used in evidence synthesis for systematic reviews, the authors could consider discussing this with their academic librarian regarding their scoping review.

Is the study design appropriate for the research question?

Yes

Is the rationale for, and objectives of, the study clearly described?

Yes

Are sufficient details of the methods provided to allow replication by others?

Yes

Are the datasets clearly presented in a useable and accessible format?

Yes

Reviewer Expertise:

Food literacy and nutrition literacy, adolescent health, systematic reviews, narrative reviews, qualitative research, quantitative research, mixed methods

I confirm that I have read this submission and believe that I have an appropriate level of expertise to confirm that it is of an acceptable scientific standard.

## References

[1] : The Role of Google Scholar in Evidence Reviews and Its Applicability to Grey Literature Searching. *PLoS One* .2015;10(9) : 10.1371/journal.pone.0138237 e0138237 10.1371/journal.pone.0138237 26379270PMC4574933
